# The Bunkie Test: Descriptive Data for a Novel Test of Core Muscular Endurance

**DOI:** 10.1155/2015/780127

**Published:** 2015-02-11

**Authors:** Jason Brumitt

**Affiliations:** George Fox University, 414 N. Meridian Street, #V123, Newberg, OR 97132, USA

## Abstract

The Bunkie test, a functional performance test consisting of 5 test positions (performed bilaterally), has been used to assess aspects of muscular function. Current performance measures are based on clinical recommendations. The purpose of this study was to report normative data for a healthy population. One hundred and twelve subjects (mean age 25.9 ± 4.5 years) were recruited from a university setting. Subjects completed a demographic questionnaire prior to testing. Hold times for each position was measured in seconds. Subjects were able to hold many of the positions for a mean score of approximately 40 seconds. There were no side-to-side differences in test position hold times per gender. Males were able to hold some positions significantly longer than their female counterparts. Males with a lower BMI were able to hold 8 of the 10 positions significantly longer than those with a higher BMI. Bunkie test scores in subjects with a prior history of musculoskeletal injury were similar to those with no history of injury. The normative data presented in this study may be used by rehabilitation professionals when assessing and rehabilitating their patients.

## 1. Introduction

Rehabilitation professionals assess muscular endurance and strength in patients and clients utilizing a variety of tests and measures (e.g., manual muscle tests, functional performance tests, dynamometry, and isokinetic testing). Functional performance tests (FPTs) “simulate sport and activity” assessing aspects of performance, functional abilities, and/or the presence of dysfunctional movement patterns [1, 2]. FPTs (also known as functional tests) have gained popularity for assessing risk of injury, identifying dysfunction, tracking progress during a rehabilitation program, and clearing an athlete to return to sport [3–7]. Rehabilitation professionals utilize FPTs to assess muscular endurance or strength in patients and clients that cannot be easily assessed with other clinical tests [2, 8, 9].

Assessing muscular endurance of the core (e.g., lumbopelvic musculature) is frequently performed by having a patient or client assume a static posture recording how long one can maintain the position. McGill et al. have described three FPTs to assess muscular endurance capacity of the core: the lateral musculature test (performed bilaterally), the flexor endurance test, and the back extensors test [10, 11]. Each test is timed to identify one's muscular endurance capacity. The relationship between test scores is calculated to identify individuals who may be at risk for a low back injury [11]. Schellenberg et al. reported mean hold durations for the prone and supine bridge tests in asymptomatic individuals and those with low back pain (LBP) [12]. Asymptomatic subjects were able to hold the prone bridge (72.5 ± 32.6 s) and the supine bridge (170.4 ± 42.5 s) significantly longer than those with LBP (prone bridge = 28.3 ± 26.8 s; supine bridge 76.7 ± 48.9 s) [12]. The FPT scores for the core musculature are used by rehabilitation professionals to guide therapeutic exercise prescription [11, 13].

de Witt and Venter have proposed a FPT consisting of 5 test positions (each test performed bilaterally for a total of 10 tests) which requires the subject to assume plank or modified plank postures with one's lower extremities supported on a bench [14]. This FPT has been coined the Bunkie test, derived from “bankie” the Afrikaans word for a little bench [14]. de Witt and Venter suggest that endurance athletes should be able to hold each test position for up to 40 s [14]. The aforementioned value represents a clinical recommendation by de Witt and Venter [14]; however, normative values for a general population are currently unknown.

The purpose of this investigation was to present normative data for the Bunkie test in a healthy, general (noncompetitive athlete) population. It was hypothesized that there would be no statistical difference in Bunkie test scores between sides (e.g., right side versus left side) within each gender group, between genders, or per demographic characteristic. It was also hypothesized that there would be a statistical difference in scores based on history of musculoskeletal injury.

## 2. Materials and Methods

### 2.1. Subjects

One hundred and twelve subjects (81 females, mean age 25.9 ± 4.4 y; 31 males, mean age 26.1 ± 4.7 y) were recruited from a university graduate school setting. Subjects were recruited to participate in the study either via direct invitation or via recruitment flyers distributed throughout the university. A subject was excluded from testing if she/he was under the age of 18, was a female who was pregnant, was currently experiencing musculoskeletal pain, or was currently receiving treatment for musculoskeletal symptoms from a licensed medical professional (e.g., medical doctor or other primary care provider, physical therapist, or chiropractor). The Institutional Review Board of Pacific University (Forest Grove, OR) approved this study.

Each subject completed a brief questionnaire collecting demographic information including age, gender, previous injuries to the extremities that required medical care (from a primary provider or allied health care provider), and previous injuries to the spine or pelvis that required medical care (from either a primary provider or allied health care provider). Height (to nearest half inch) and weight (to nearest half pound) were recorded using a standard medical scale.

### 2.2. Procedure

**Figure 1:**
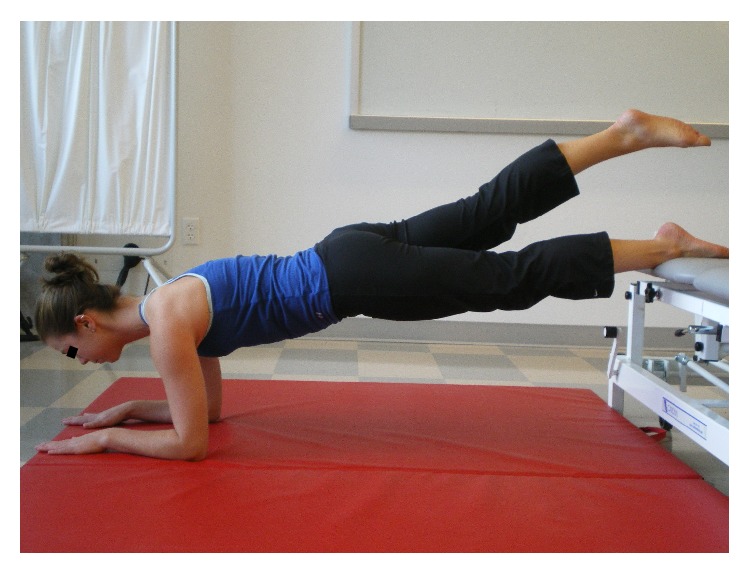
Anterior power line (APL).

**Figure 2 fig2:**
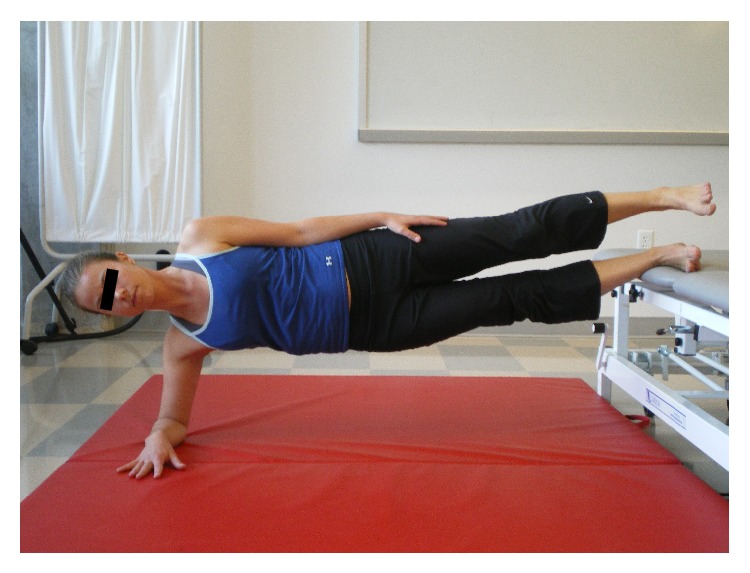
Lateral stabilizing line (LSL).

**Figure 3 fig3:**
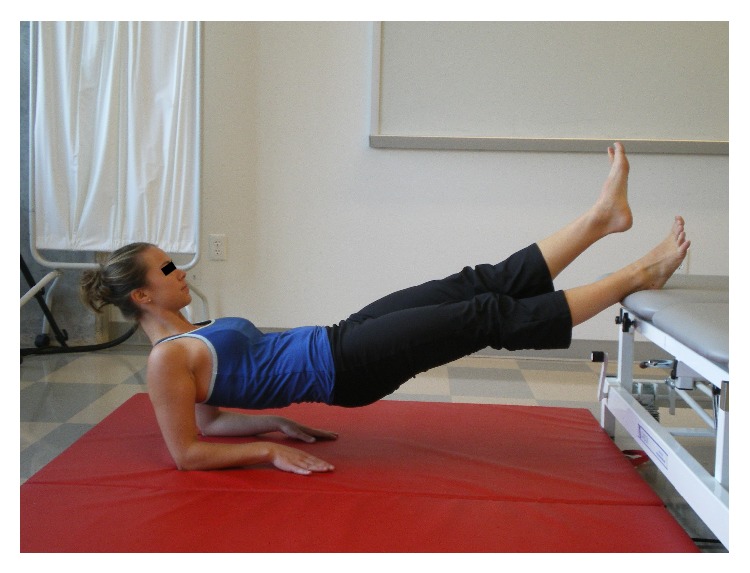
Posterior power line (PPL).

**Figure 4 fig4:**
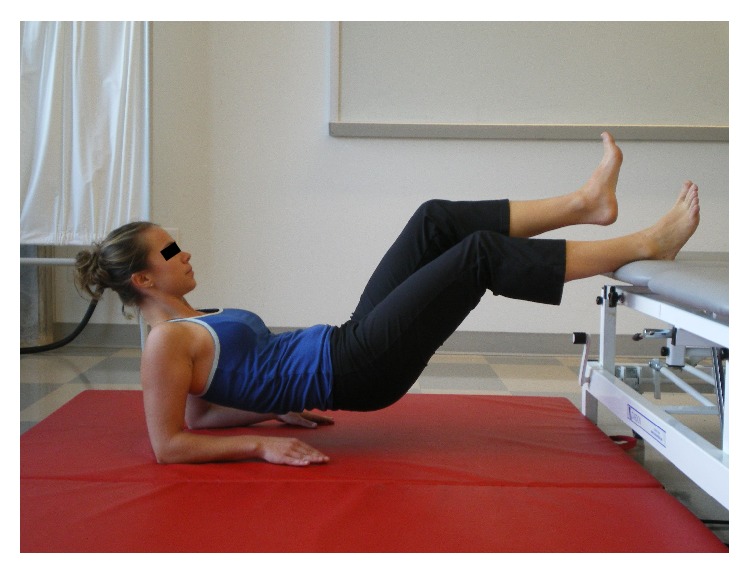
Posterior stabilizing line (PSL).

**Figure 5:**
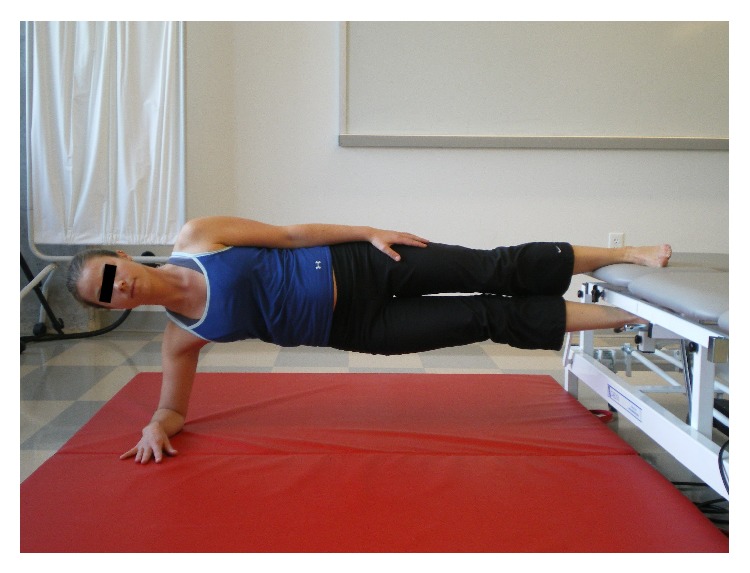
Medial stabilizing line (MSL).

The Bunkie test consists of 5 testing positions with each test performed bilaterally (Figures 1 [Fig fig2] [Fig fig3] [Fig fig4]–5). Order of testing was randomized per each subject. A roll of a die determined order of testing (roll of 1 = anterior power line (APL); 2 = lateral stabilizing line (LSL); 3 = posterior power line (PPL); 4 = posterior stabilizing line (PSL); 5 = medial stabilizing line (MSL); 6 = roll again). Sequencing of the remaining tests was based on the initial number rolled. For example, a subject who rolled a 4 would perform the PSL first with the remaining tests performed sequentially (5, 1, 2, 3). A flip of a coin was performed to determine which side was tested first.

Subjects were shown a picture of each test (see Figures 1 [Fig fig2] [Fig fig3] [Fig fig4]–5) and asked to assume the test position with their upper extremities placed against a floor mat and the lower extremities (LE) positioned (approximately mid-Achilles) on the treatment table. The height between the top of the mat and the treatment table top was standardized at 30 cm. Once in position, the primary investigator (PI) provided verbal cues to help facilitate the correct posture prior to initiating the test. The PI next instructed the subject to elevate one LE off of the surface of the treatment table. For this study, when the right LE was weight-bearing on the treatment table it was described as a right sided test. The time that one was able to maintain the proper test position was recorded in seconds using a stopwatch. A test was terminated when a subject was no longer able to maintain the proper test position (as shown in Figures 1 [Fig fig2] [Fig fig3] [Fig fig4]–5). Examples of test termination occurred when either (a) the subject stopped the test due to fatigue or (b) the subject was unable to maintain the correct position. Subjects were allowed one attempt to correct their position; if they were unable to assume the correct posture after verbal cueing the test was stopped. Thirty seconds of rest was allowed between tests.

### 2.3. Statistical Analysis

Means (±SD) were calculated for age, height, weight, BMI, and hold times for each Bunkie test position. Independent* t*-tests were calculated to assess for differences in hold times between lower extremities for each group (all subjects, females, males). Independent* t-*tests were calculated to assess for differences in test scores based on demographic characteristics: mean age, mean BMI, and prior history of musculoskeletal injury. Independent* t*-tests were also calculated to assess for differences in Bunkie test position hold times between genders. Data analyses were performed using SPSS 17.0 with alpha level set at 0.05.

## 3. Results and Discussion

The test-retest reliability for each position was calculated during a pilot study prior to subject recruitment. The intraclass correlation coefficients (ICC_3,1_) were as follows: APL was 0.82 (95% CI: 0.67, 0.94); LSL was 0.95 (95% CI: 0.90, 0.98); PPL was 0.95 (95% CI: 0.90, 0.98), PSL was 0.92 (95% CI: 0.84, 0.97), and the MSL was 0.95 (95% CI: 0.90, 0.98).

Demographic information of the study sample is presented in Table 1. Eighty-one of the 112 subjects were female. Fifty-three of the 81 female subjects (65 percent) reported a prior history of musculoskeletal injury that required evaluation and treatment by a medical professional. Twenty-one of the 81 female subjects (26 percent) reported history of back (thoracic or lumbar region) injury that required evaluation and treatment by a medical professional. Sixty-four percent (20 out of 31) of male subjects reported prior history of musculoskeletal injury requiring medical treatment. Only 13 percent (4 out of 31) of male subjects reported prior history of back (thoracic or lumbar region) injury.

Mean (± SD) Bunkie scores for the 5 tests (10 positions) are presented in Table 2. “All subjects” (e.g., both female and male subjects) were able to hold 4 of the test positions for at least a minimum of 40 s (mean score): APL (L), LSL (R), PPL (R), and PPL (L). Mean scores for 4 other test positions, APL (R), LSL (L), PSL (R), and PSL (L), were very close to 40 s. The mean hold times for the MSL tests (R = 23.6 (±15.0) s; L = 22.2 (±13.9) s) were shorter in duration when compared to all other test positions. There were no side-to-side differences between extremities for the total population.

Female subjects were able to hold three test positions for a mean score of a minimum of 40 s (mean score): LSL (R), PPL (R), and the PPL (L) (Table 2). Females held the MSL position for the shortest time period (R = 21.3 (±15.5) s; L = 20.3 (±13.9) s). Female subjects were able to hold the PPL for the longest time period (R = 46.9 (±21.6) s; L = 50.3 (±24.6) s). There were no side-to-side differences between lower extremities for each test position in this group.

Male subjects were able to hold five test positions for a minimum of 40 s (mean score): APL (R), APL (L), LSL (R), PPL (R), and the PPL (L) (Table 2). Males held the MSL position for the shortest time period (R = 29.8 (±11.9) s; L = 27.0 (±13.1) s). Male subjects were able to hold the PPL for the longest time period (R = 46.3 (±23.7) s; L = 46.5 (±31.4) s). There were no side-to-side differences between lower extremities for each test position in this group.

Male subjects held three of the test positions significantly longer than their female counterparts. Males were able to hold the APL (L) position for 45.5 (±17.5) s whereas females were only able to hold this test position 37.9 (±14.2) s (*P* = 0.02) (Table 2). Males were also able to hold each MSL test position (R = 29.8 (±11.9) s; L = 27.0 (±13.1) s) significantly longer than their female counterparts (R = 21.3 (±15.5) s, *P* = 0.007; L = 20.3 (±13.9) s, *P* = 0.02).

Table 3 presents Bunkie test scores for “all subjects” based on age, BMI, and prior history of injury. Age and BMI were categorized by this study's population mean scores. There were no significant differences between Bunkie test scores based on one's age. There were two significant findings based on BMI. Those with a lower BMI (<23 kg/m^2^) were able to hold the PSL test position significantly longer (R *P* = 0.04; L *P* = 0.05) than those with a greater BMI (≥23 kg/m^2^). Those with a lower BMI were also able to hold the (R) PSL position significantly longer than their counterparts in the higher BMI category (*P* = 0.04). There was only one test position, the LSL (L), where a significant difference in hold times was observed between those with no history or history of musculoskeletal injury. Those with a history of injury were able to hold the LSL (L) position 42.1 (±18.4) s whereas those with no history of musculoskeletal pain only held the position for 34.6 (±15.9) s (*P* = 0.03).

Table 4 presents Bunkie test scores for female subjects based on age, BMI, and prior history of musculoskeletal injury. Younger female subjects were able to hold the PSL (L) test position (41.6 ± 18.1 s) significantly longer than older female subjects (33.2 ± 14.6 s) (*P* = 0.05). Female subjects with a lower BMI were able to hold the PPL test position on the right significantly longer than those with a larger BMI (*P* = 0.02). There were no statistical differences in hold times when comparing female subjects with or without history of musculoskeletal injury.

Table 5 presents Bunkie test scores for male subjects based on age, BMI, and prior history of injury. There were no statistical differences between test scores for male subjects based on age categorization. Male subjects with a lower BMI were able to hold 8 out of the 10 test positions significantly longer than males with a greater BMI. There were no statistical differences in hold times when comparing male subjects with or without history of musculoskeletal injury.

This is the first study to report normative Bunkie test scores for a healthy, general (noncompetitive athlete) population. In general, subjects were able to hold test positions for mean times of approximately 40 seconds except for the MSL test position. There were no within group side-to-side differences per gender; however, males were able to hold some positions significantly longer than their female counterparts. There were occasional significant differences in test scores based on age and BMI in the “all subjects” and female groups. These significant differences should be viewed as preliminary and may be the result of the population sampled in this study. Male subjects with a lower BMI were able to hold the test positions significantly longer in most cases than males with a greater BMI. This finding is opposite of what was observed with female subjects. It is possible that males with a higher BMI were less conditioned than their counterparts with a lower BMI; however, this is only speculative. The relationship between higher BMI and lower Bunkie scores warrants further exploration.

Part of the process of determining a test's utility is to identify normative values. McGill [11] published descriptive data for mean endurance times for the lateral musculature test, the flexor endurance test, and the back extensors test in 21-year-old asymptomatic individuals. Women were able to hold the back extensor test for a longer period than the males; however, the males were able to hold the other 3 positions for longer periods [11]. In this study, the males were able to hold some of the Bunkie test positions longer than their female counterparts. McGill [11] also reported that asymptomatic individuals with a history of LBP have shorter hold times with the muscular endurance tests for the core and have abnormal ratios between tests. The muscular dysfunction in individuals with prior history of LBP is consistent with the finding that muscular function of the multifidus is not spontaneous after an initial episode of LBP [15]. In this study's healthy population, the Bunkie test did not differentiate between those with or without prior musculoskeletal injury. This is counter to what was hypothesized. When comparing subjects' Bunkie scores based on prior history of injury (with either a prior history of musculoskeletal injury or only history of prior back injury) there was only one significant relationship observed. Interestingly, in the “all subjects” group, those who reported a prior history of musculoskeletal injury held the LSL (L) significantly longer than those who had no prior history.

The testing protocol for the Bunkie test in this investigation differs slightly from the original description by de Witt and Venter [14]. de Witt and Venter [14] described the Bunkie test as a tool to assess fascial mobility. They proposed that each of the 5 test positions assesses different fascial planes [14]. According to de Witt and Venter [14], the tests are held for specific time periods assessing the patient or client for symptoms such as pain, cramping, or burning. The occurrence of these symptoms within the 20 to 40 s testing range (40 s for endurance athletes and less time for general population) is purported to identify fascial dysfunction [14]. However, assessment and treatment of fascial dysfunction are not accepted amongst all physical therapists or other rehabilitation professionals. More research is warranted to validate the presence of functional fascial planes and execution of randomized controlled trials to assess outcomes of treatment directed at fascial dysfunction. In addition, musculoskeletal sensations such as “cramping,” “burning,” and “pain” are subjective experiences and currently not correlated with dysfunction in asymptomatic subjects. For this study, instead of terminating a test based on the patient's subjective musculoskeletal experience the primary investigator (PI) recorded how long a subject could hold a test position with correct technique. Interestingly, the subjects were able to hold a majority of test positions for mean times of approximately 40 s except for the MSL positions.

Brumitt demonstrated how the Bunkie test could be utilized as a measure of core muscular endurance capacity in the assessment of an injured recreational distance runner [16]. The patient, a 24-year-old female, experienced left-sided low back pain only when running. Testing the patient with the Bunkie revealed asymmetrical hold times that correlated with left-sided weakness of the gluteus maximus, gluteus medius, and hip external rotators (as assessed by traditional manual muscle testing) [16]. The prescription of therapeutic exercises targeting core muscular weakness improved the patient's ability to activate her gluteal muscles. At her follow-up visit 8 days later she was able to hold the Bunkie test positions for longer periods and return to running pain-free.

One other study to date has reported mean scores for the Bunkie test. van Pletzen and Venter reported Bunkie scores in 121 elite-level rugby union athletes [17]. Mean scores for front row rugby players ranged from the lowest score of 21.51 (±12.56) s for the medial stabilizing line (left side) to the highest score of 35.63 (±9.21) s for the anterior power line (right side). Mean scores for backline rugby players range from the lowest score of 27.96 (±13.77) s for the posterior stabilizing line (left side) to the highest score of 39.87 (±0.66) s for the anterior power line (right side). van Pletzen and Venter [17] utilized a similar testing protocol as that described by de Witt and Venter [14] having the rugby player hold the test position up to a maximum of 40 s. Despite the time restriction and test termination requirement based on musculoskeletal sensations, the subjects in van Pletzen and Venter [17] held the tests for similar time periods as subjects in this study.

Future investigations are warranted to determine the utility of the Bunkie test. Descriptive studies are warranted to identify normative data in injured populations (e.g., chronic low back). In this study subjects with a prior history of injury did not demonstrate significant differences in hold times when compared to individuals with no history of injury. However, for an injured patient the Bunkie test may be clinically useful as a test to identify asymmetry of muscular endurance or as a tool to track increases in muscular function during a course of rehabilitation [16]. Clinicians should utilize caution and their clinical judgment when assessing the injured patient. The Bunkie test may be too aggressive for patients who are in the acute stage of healing; however, those who are in subacute or chronic stages may be able to tolerate the test without symptom provocation. The Bunkie test should also be evaluated for its ability to identify individuals who may be at risk for a future injury (e.g., endurance athletes, manual laborers). For example, the test should be administered to a cohort of athletes at the start of the season with scores assessed at the end of the season to determine if associations exist between time-loss injuries and preseason performance. Finally, aspects of the testing protocol warrant further assessment. In this study, the height of the table top to the floor was standardized for all subjects to 30 cm. de Witt and Venter [14] recommended a range of 25 to 30 cm depending on individual size; however, no guidance was provided as to a how to set the Bunkie floor-to-bench height based on an individual's height. In this study subjects were allowed 30 seconds of rest between each test. The 30 s time period was selected to replicate how some FPTs are administered clinically [2]. What length of rest should be allowed to optimize recovery is yet to be determined.

## 4. Conclusion

This investigation presents normative data for the Bunkie test in healthy individuals. Subjects in this study were able to hold many of the positions for a mean score of approximately 40 seconds. There were no side-to-side differences in test position hold times per gender. For the most part, Bunkie test scores were similar between those with prior history of musculoskeletal injury and those with no prior history. This normative data may be useful for rehabilitation professionals when comparing their patient's or client's Bunkie test scores to a general population.

## Figures and Tables

**Table 1:** Demographic characteristics (mean ± SD).

Characteristic	Total (*n* = 112)	Females (*n* = 81)	Males (*n* = 31)
Age (y)	25.9 (4.5)	25.9 (4.4)	26.1 (4.7)
Height (m)	1.69 (.09)	1.66 (.06)	1.79 (.07)
Weight (kg)	66.8 (12.5)	61.2 (6.8)	81.3 (12.3)
BMI (kg/m^2^)	23.2 (3.0)	22.3 (2.3)	25.5 (3.4)
Prior history of musculoskeletal injury (prior history of injury/total *N*)	73/112	53/81	20/31
Prior history of back injury (prior history of injury/total *N*)	25/112	21/81	4/31

**Table 2:** Bunkie test scores (mean ± SD) and comparisons between female and male subjects.

Bunkie test position (seconds)	Total (*n* = 112)	*P* value^*^	Females (*n* = 81)	*P* value^*^	Males (*n* = 31)	*P* value^*^	Between gender differences *P* value^*^
APL							
R	38.6 (16.3)	0.5	36.9 (16.7)	0.7	42.9 (14.5)	0.5	0.09
L	40.0 (15.5)		37.9 (14.2)		45.5 (17.5)		**0.02**
LSL							
R	42.0 (18.8)	0.3	41.9 (20.4)	0.5	42.1 (14.1)	0.3	0.9
L	39.5 (17.9)		39.9 (19.4)		38.6 (13.5)		0.7
PPL							
R	46.7 (22.1)	0.4	46.9 (21.6)	0.3	46.3 (23.7)	0.9	0.9
L	49.2 (26.6)		50.3 (24.6)		46.5 (31.4)		0.5
PSL							
R	38.6 (17.3)	0.9	38.7 (17.3)	0.9	38.1 (17.5)	0.7	0.9
L	38.4 (17.6)		39.0 (17.4)		36.7 (18.0)		0.5
MSL							
R	23.6 (15.0)	0.4	21.3 (15.5)	0.7	29.8 (11.9)	0.4	**0.007**
L	22.2 (13.9)		20.3 (13.9)		27.0 (13.1)		**0.02**

^*^Independent *t*-tests.

APL = anterior power line; LSL = lateral stabilizing line; PPL = posterior power line; PSL = posterior stabilizing line; MSL = medial stabilizing line.

**Table 3:** Bunkie test scores (mean ± SD) for all subjects (*n* = 112) based on age, BMI, and prior history of injury.

Bunkie test position (seconds)	Age <26 y (*n* = 75)	Age ≥26 y (*n* = 37)	*P* ^*^	BMI <23 (*n* = 55)	BMI ≥23 (*n* = 57)	*P* ^*^	History of prior musculoskeletal injury		*P* ^*^	History of prior back injury		*P* ^*^
							Yes (*n* = 73)	No (*n* = 39)		Yes (*n* = 25)	No (*n* = 87)	
APL												
R	38.4 (17.7)	39.1 (13.0)	0.8	40.9 (17.4)	36.4 (14.9)	0.1	38.1 (16.0)	39.5 (16.9)	0.7	36.9 (17.8)	39.1 (15.9)	0.6
L	39.5 (15.9)	41.1 (14.9)	0.6	41.3 (16.6)	38.8 (14.4)	0.4	41.2 (16.4)	37.8 (13.6)	0.3	36.7 (15.8)	41.0 (15.4)	0.2
LSL												
R	39.6 (20.4)	46.8 (14.0)	0.06	42.4 (17.9)	41.6 (19.7)	0.8	41.9 (19.3)	42.1 (18.0)	0.9	41.5 (22.9)	42.1 (17.6)	0.9
L	37.5 (18.6)	43.6 (15.8)	0.09	40.4 (18.5)	38.6 (17.4)	0.6	42.1 (18.4)	34.6 (15.9)	**0.03**	43.5 (20.5)	38.4 (17.0)	0.2
PPL												
R	45.7 (22.0)	48.8 (22.3)	0.5	50.7 (20.1)	42.9 (23.4)	0.06	47.6 (23.2)	45.2 (19.9)	0.6	46.3 (21.9)	46.9 (22.2)	0.9
L	46.4 (25.5)	55.0 (28.1)	0.1	53.4 (23.9)	45.2 (28.6)	0.1	52.0 (30.2)	44.2 (17.1)	0.1	50.0 (29.3)	49.0 (25.9)	0.9
PSL												
R	38.8 (17.5)	38.1 (17.0)	0.8	42.1 (17.3)	35.2 (16.7)	**0.04**	38.2 (17.5)	39.2 (17.0)	0.8	38.9 (19.6)	38.5 (16.7)	0.9
L	39.4 (18.1)	36.3 (16.4)	0.4	41.6 (16.7)	35.2 (17.9)	**0.05**	37.1 (16.4)	40.7 (19.5)	0.3	37.8 (18.3)	38.5 (17.4)	0.8
MSL												
R	23.6 (15.5)	23.6 (14.1)	0.9	25.3 (12.9)	22.1 (16.8)	0.3	23.0 (16.0)	24.7 (13.2)	0.6	19.2 (13.2)	24.9 (15.4)	0.1
L	22.0 (14.4)	22.6 (13.1)	0.8	23.7 (12.0)	20.7 (15.6)	0.3	22.6 (14.8)	21.3 (12.2)	0.7	20.4 (12.9)	22.7 (14.2)	0.5

^*^Independent *t*-tests.

APL = anterior power line; LSL = lateral stabilizing line; PPL = posterior power line; PSL = posterior stabilizing line; MSL = medial stabilizing line.

**Table 4:** Bunkie test scores (mean ± SD) for female subjects (*n* = 81) based on age, BMI, and prior history of injury.

Bunkie test position (seconds)	Age< 26 y (*n* = 56)	Age ≥26 y (*n* = 25)	*P* ^*^	BMI <22 (*n* = 41)	BMI ≥22 (*n* = 40)	*P* ^*^	History of prior musculoskeletal injury		*P* ^*^	History of prior back injury		*P* ^*^
							Yes (*n* = 53)	No (*n* = 28)		Yes (*n* = 21)	No (*n* = 60)	
APL												
R	38.2 (18.8)	34.3 (10.5)	0.3	39.1 (18.3)	34.8 (14.8)	0.4	36.4 (16.8)	38.1 (16.7)	0.7	35.4 (18.7)	37.5 (16.1)	0.6
L	38.7 (15.6)	36.2 (10.5)	0.5	39.0 (16.5)	36.8 (11.6)	0.5	38.9 (15.7)	36.0 (10.9)	0.4	34.4 (13.7)	39.1 (14.3)	0.2
LSL												
R	39.7 (22.0)	46.8 (15.3)	0.1	42.0 (19.6)	41.8 (21.4)	0.9	41.7 (20.9)	42.3 (19.6)	0.9	42.4 (24.6)	41.7 (18.9)	0.9
L	38.1 (20.0)	43.9 (17.8)	0.2	40.8 (18.7)	38.9 (20.3)	0.7	42.5 (20.0)	34.8 (17.3)	0.1	42.8 (22.3)	38.9 (18.4)	0.4
PPL												
R	47.6 (21.9)	45.3 (21.1)	0.7	52.5 (19.6)	41.1 (22.2)	**0.02**	47.3 (22.0)	46.1 (21.2)	0.8	45.3 (23.1)	47.4 (21.2)	0.7
L	49.3 (25.5)	52.7 (22.5)	0.6	54.8 (22.6)	45.8 (25.9)	0.1	52.6 (27.5)	46.0 (17.3)	0.3	49.6 (31.6)	50.6 (21.9)	0.9
PSL												
R	40.5 (17.8)	34.8 (15.8)	0.2	42.2 (17.9)	35.2 (16.2)	0.07	39.3 (17.5)	37.6 (17.2)	0.7	40.3 (20.6)	38.2 (16.2)	0.6
L	41.6 (18.1)	33.2 (14.6)	**0.05**	42.5 (17.7)	35.4 (16.6)	0.07	37.8 (16.3)	41.4 (19.5)	0.4	38.7 (19.6)	39.1 (16.8)	0.9
MSL												
R	22.1 (16.8)	19.4 (12.4)	0.5	22.6 (12.0)	19.9 (18.5)	0.4	21.2 (16.7)	21.4 (13.3)	0.9	16.5 (10.9)	23.0 (16.6)	0.1
L	20.7 (14.8)	19.3 (11.8)	0.7	21.9 (11.5)	18.7 (15.9)	0.3	20.7 (14.4)	19.5 (13.0)	0.7	17.7 (10.6)	21.2 (14.8)	0.3

^*^Independent *t*-tests.

APL = anterior power line; LSL = lateral stabilizing line; PPL = posterior power line; PSL = posterior stabilizing line; MSL = medial stabilizing line.

**Table 5:** Bunkie test scores (mean ± SD) for male subjects (*n* = 31) based on age, BMI, and prior history of injury.

Bunkie test position (seconds)	Age <26 y (*n* = 19)	Age ≥26 y (*n* = 12)	*P* ^*^	BMI <25 (*n* = 17)	BMI ≥25 (*n* = 14)	*P* ^*^	History of prior musculoskeletal injury		*P* ^*^	History of prior back injury		*P* ^*^
							Yes (*n* = 20)	No (*n* = 11)		Yes (*n* = 4)	No (*n* = 27)	
APL												
R	39.0 (14.7)	49.1 (12.3)	0.06	47.4 (15.3)	37.4 (11.7)	**0.05**	42.8 (12.9)	43.0 (17.8)	0.9	44.8 (10.1)	42.6 (15.2)	0.8
L	41.8 (16.8)	51.3 (17.8)	0.15	54.8 (16.9)	34.3 (10.6)	**0.00**	47.2 (17.1)	42.5 (18.7)	0.5	48.8 (22.9)	45.0 (17.1)	0.7
LSL												
R	39.3 (15.1)	46.6 (11.6)	0.2	44.7 (15.5)	38.9 (12.0)	0.3	42.3 (14.5)	41.7 (14.0)	0.9	36.5 (10.6)	42.9 (14.5)	0.4
L	35.8 (14.2)	43.1 (11.4)	0.15	38.2 (14.3)	39.1 (13.0)	0.9	41.1 (13.7)	34.2 (12.5)	0.2	47.5 (2.9)	37.3 (14.0)	0.2
PPL												
R	40.2 (22.1)	56.0 (23.8)	0.07	57.1 (22.1)	33.2 (19.0)	**0.003**	48.4 (26.9)	42.6 (17.1)	0.5	51.3 (16.0)	45.6 (24.8)	0.7
L	38.1 (23.9)	59.7 (38.1)	0.06	61.5 (32.7)	28.2 (17.8)	**0.002**	50.4 (37.1)	39.4 (16.1)	0.4	51.8 (14.1)	45.7 (33.4)	0.7
PSL												
R	33.8 (16.1)	44.9 (18.1)	0.08	45.2 (14.7)	29.6 (17.2)	**0.01**	35.4 (17.6)	43.1 (16.9)	0.3	31.5 (12.5)	39.1 (18.1)	0.4
L	32.9 (17.0)	42.7 (18.7)	0.1	44.1 (15.7)	27.6 (16.8)	**0.01**	35.4 (17.0)	39.1 (20.3)	0.6	32.8 (8.4)	37.3 (19.1)	0.6
MSL												
R	28.1 (10.4)	32.4 (14.0)	0.3	33.7 (12.6)	25.0 (9.3)	**0.04**	27.9 (13.2)	33.3 (8.4)	0.2	33.3 (17.0)	29.3 (11.3)	0.5
L	25.5 (12.9)	29.4 (13.5)	0.4	31.5 (13.7)	21.6 (10.2)	**0.03**	27.7 (15.0)	25.8 (9.1)	0.7	34.3 (16.7)	26.0 (12.5)	0.2

^*^Independent *t*-tests.

APL = anterior power line; LSL = lateral stabilizing line; PPL = posterior power line; PSL = posterior stabilizing line; MSL = medial stabilizing line.
